# *Miniphocibacter massiliensis* gen. nov., sp. nov., a new species isolated from the human gut and its taxono‐genomics description

**DOI:** 10.1002/mbo3.735

**Published:** 2018-10-02

**Authors:** Melhem Bilen, Maxime D. Mbogning Fonkou, Thi T. Nguyen, Magali Richez, Ziad Daoud, Pierre E. Fournier, Didier Raoult, Frédéric Cadoret

**Affiliations:** ^1^ Aix Marseille Univ IRD, APHM MEPHI IHU‐Méditerranée Infection Marseille France; ^2^ Clinical Microbiology Department Faculty of Medicine and Medical Sciences University of Balamand Amioun Lebanon; ^3^ Special Infectious Agents Unit King Fahd Medical Research Center King Abdulaziz University Jeddah Saudi Arabia

**Keywords:** culturomics, gut microbiota, *Miniphocibacter massiliensis*, new species, pygmy, taxono‐genomics

## Abstract

With the aim of describing the human microbiota by the means of culture methods, culturomics was developed in order to target previously un‐isolated bacterial species and describe it via the taxono‐genomics approach. While performing a descriptive study of the human gut microbiota of the pygmy people, strain Marseille‐P4678^T^ has been isolated from a stool sample of a healthy 39‐year‐old pygmy male. Cells of this strain were Gram‐positive cocci, spore‐forming, non‐motile, catalase‐positive and oxidase‐negative, and grow optimally at 37°C under anaerobic conditions. Its 16S rRNA gene sequence exhibited 89.69% of sequence similarity with *Parvimonas micra* strain 3119B^T^ (NR 036934.1), its phylogenetically closest species with standing in nomenclature. The genome of strain Marseille‐P4678^T^ is 2,083,161 long with 28.26 mol% of G+C content. Based on its phenotypic, biochemical, genotypic and proteomic profile, this bacterium was classified as a new bacterial genus and species *Miniphocibacter massiliensis* gen. nov., sp. nov. with the type strain Marseille‐P4678^T^.

## 1 INTRODUCTION

Ever since it has been proved to play a role in the human health and diseases, the human gut microbiota took more attention and descriptive studies have been exhaustively done toward unveiling its microbial content (Clemente, Ursell, Parfrey, & Knight, 2012). For example, the gut microbiota composition has been shown to play a role in malnutrition, Crohn's disease, inflammatory bowel diseases, etc. and thus became a therapeutic target in several cases (Million et al., 2016; Rigottier‐Gois, 2013; Tidjani Alou et al., 2017; Vétizou et al., 2015; Würdemann et al., 2009). Yet, with the expansion of culture‐independent techniques, scientists’ thought that the human microbiota could be more efficiently described with no need of re‐adapting culture approaches. Indeed, with metagenomics, loads of data have been reported out of which some were significant and others were not since several drawbacks can be faced throughout the process such as the depth bias, presence of operational taxonomic units (OTUs) inability to identify certain pathogenic species or distinguish dead from living bacteria, and thus rendering a part of the human gut population neglected, not identified or described (Greub, 2012). Thus, culturomics was developed, based on sophisticated culture methods, targeting previously uncultured bacterial species (Lagier et al., 2012). The latter was able to report a significant number of nonhuman and new bacterial species along with correlating its sequences to different OTUs (Lagier et al., 2016). New bacterial species are described with the means of taxono‐genomics approach, which relies on characterizing the understudied organism at the phenotypic, biochemical, proteomic and genomic level in order to confirm the novelty of the understudied organism whenever full genomes are available for comparison at wider range (Fournier & Drancourt, 2015; Lagier et al., 2016). Knowing that 1g of human stool might contain up to 1012 bacterial cells and that only around 2,000 species were isolated by culture, motivate us to purse our efforts in describing the human gut microbiota by culturomics. (Hugon et al., 2015; Raoult & Henrissat, 2014). Herein, we describe a new bacterial species, *Miniphocibacter massiliensis* gen.nov, sp. nov. strain Marseille‐P4678^T^ using the taxono‐genomics approach. This bacterium was isolated from the stool samples of a healthy pygmy male.

## 2 MATERIAL AND METHODS

### 2.1 Strain Marseille‐P4678^T^ isolation and identification

Shipment of the stool samples was done using a special medium C‐Top Ae‐Ana (Culture Top, Marseille, France) and stored at our laboratory at ‐80°C for further analysis. To assess bacterial content using culturomics, the stool sample was diluted with phosphate buffer saline and incubated in an anaerobic culture bottles (BD BACTEC^®^, Plus Anaerobic/F Media, Le Pont de Claix, France) supplemented with 5% (V/V) sheep blood and 5% (V/V) sterile‐filtered cow rumen at 37°C. Subculturing assays were done on 5% sheep blood–enriched Columbia agar (bioMérieux, Marcy l'Etoile, France) for bacterial colonies isolation. Isolated colonies were identified using MALDI‐TOF MS (matrix‐assisted laser desorption ionization time of flight mass spectrometry; Microflex Lt [Bruker Daltonics, Bremen, Germany]) as previously described (Lagier et al., 2012; Seng et al., 2010). Whenever MALDI‐TOF MS fails to identify the tested organism, 16S rRNA gene sequencing was performed as formerly done for further phylogenetic analysis (Drancourt, Berger, & Raoult, 2004). CodonCode Aligner tool (http://www.codoncode.com) served for sequence optimization and alignment. Retrieved sequences were blasted in NCBI nucleotide database (http://blast.ncbi.nlm.nih.gov.gate1.inist.fr/Blast.cgi), and species or genera delimitation thresholds were adapted according to Kim, Oh, Park, and Chun (2014) norms. EMBL‐EBI (https://www.ebi.ac.uk/services) and UMRS (http://www.mediterranee infection.com/article.php?laref=256&titre=urms‐database) databases were used for 16S rRNA gene sequence and mass spectrum deposition, respectively.

### 2.2 Optimal growth conditions

Several growth attempts were done with various culture conditions in order to determine the optimal growth of strain Marseille‐P4678^T^. The following criteria were taken into consideration: pH (6, 6.5, 7, and 8.5), temperatures (25, 37, 45, and 55°C), NaCl concentrations (0, 5, 10, 50, 75, and 100 g/L), and atmosphere (anaerobic (GENbag anaer, bioMérieux, France), microaerophilic (GENbag Microaer, bioMérieux, France), and aerobic).

### 2.3 Biochemical, antibiotic resistance, and phenotypic characteristics of strain Marseille‐P4678^T^

To biochemically describe strain Marseille‐P4678^T^, API strips (ZYM, 20A and 50CH; bioMérieux) were used according to manufacturer's protocol. Sporulation ability was tested by performing a culture assay of a previously heat‐shocked bacterial suspension (80°C; 20 min). Gram staining and motility were evaluated using a DM1000 photonic microscope (Leica Microsystems, Nanterre, France) at 100 ×  oil immersion lens, and cell morphology was determined using an electron microscope as formerly done (Bilen et al., 2018).

Additionally, to test the antibiotic resistance characteristics of strain Marseille‐P4678^T^, several E‐strips were used: voriconazole, vancomycin, tobramycin, teicoplanin, rifampicin, minocycline, metronidazole, kanamycin, imipenem, fosfomycin, fluconazole, erythromycin, ertapenem, daptomycin, colistin, ceftriaxone, benzylpenicillin, amoxycillin, amikacin (bioMérieux, France).

GC/MS and cellular fatty acid analysis of strain Marseille‐P4678^T^ were carried out using 20 mg (dry weight) of bacterial biomass per tube as previously done (Dione et al., 2016). SQ8s mass spectrometer (Perkin Elmer, Courtaboeuf, France) was used for short‐chain fatty acids measurements along with Clarus 500 chromatography (Zhao, Nyman, & Jönsson, 2006). Isobutyric, propionic, butyric, isovaleric, caproic, valeric, enanthic, and isocaproic (Sigma‐Aldrich; Lyon, France) were used. Calibrations were done using acidified water (pH 2–3 with HCl 37%), and SCFA were examined using three samples and three controls. Centrifugation of the culture medium was done for 5 min at 16,000 × *g* in order to get rid of the bacteria and its debris. Collected supernatant's pH was fixed between two and three and spiked with 2‐ethylbutyric acid as the internal standard (1 mm) (IS) (Sigma‐Aldrich). Centrifugation was repeated prior sample's injection. Direct injection (0.5 μl) of the aqueous solution was done in a splitless liner heated at 200°C. Injection performance was decreased with 10 syringe washes in methanol:water (50:50 v/v). Using a linear temperature gradient from 100 to 200°C at 8°C/min, compounds’ separation was done on an Elite‐FFAP column (30 m, 0.25 mm i.d., 0.25 mm film thickness). Helium flowing was used as carrier gas at 1 ml/min. MS inlet line and electron ionization source were set at 200°C. After 4.5 min, selected ion recording (SIR) was done with the following masses: 88 *m/z* (2‐ethylbutyric acid, IS), 43 *m/z* (isobutyric acid), 60 *m/z* (acetic, butyric, valeric, isovaleric, caproic, and enanthic acid), and 74 *m/z* (propanoic and isocaproic acid). All data were collected and processed using Turbomass 6.1 (Perkin Elmer, Courtaboeuf, France). The peak areas of the associated SIR chromatograms were used for quadratic internal calibration calculations. All coefficients of determination were more than 0.999. Back‐calculated standards and calculated quality controls (0.5 and 5 mm) represented accuracy with less than 15% deviation. Blank results of the control were subtracted from while analyzing samples.

### 2.4 DNA extraction and genome sequencing

Strain Marseille‐P4678^T^ genomic DNA (gDNA) was extracted by first performing acid glass beads wash (G4649‐500 g Sigma) using a FastPrep BIO 101 instrument (Qbiogene, Strasbourg, France) at maximum speed (6.5 m/s) for 90s. Two hours later, a lysozyme incubation at 37°C was done prior to DNA extraction on the EZ1 biorobot (Qiagen) with EZ1 DNA tissues kit. 50 μl gDNA of 15.8 ng/μl concentration was eluted. Concentration was measured using a Qubit assay with the high sensitivity kit (Life Technologies, Carlsbad, CA, USA). MiSeq technology (Illumina Inc, San Diego, CA, USA) was used with the mate‐pair strategy for gDNA sequencing along with 11 other projects and barcoded with the with the Nextera Mate Pair sample prep kit (Illumina). 1.5 μg of gDNA was used for library preparation according to Nextera mate‐pair Illumina guidelines. Tagmentation was done using with a mate‐pair junction adapter and simultaneously fragmented. Agilent 2100 BioAnalyzer (Agilent Technologies Inc, Santa Clara, CA, USA) was used for fragments profile validation with a DNA 7500 labchip. Fragments’ DNA sizes ranged between 1.5 and 11 kb with an optimal size at 5.933 kb. Additionally, circularization (600 ng of tagmented fragments) was performed with no size selection. The circularized DNA was mechanically sheared to small fragments with optima on a bimodal curve at 475 and 1,207 bp on the Covaris device S2 in T6 tubes (Covaris, Woburn, MA, USA). The library final concentration was measured and visualized on a High Sensitivity Bioanalyzer LabChip (Agilent Technologies Inc, Santa Clara, CA, USA) as 27.85 nmol/l. The latter was normalized at 2 nM and pooled. Denaturation was done, and a dilution step to 19 pM was performed prior to pool loading. A single 2 × 251‐bp run was performed with an automated sequencing and cluster generation method. Total information of 4.6 Gb was obtained from a 471 K/mm^2^ cluster density with a cluster passing quality control filters of 98.7% (9,100,000 passing filter paired reads). Within this run, the index representation for strain Marseille‐P4678^T^ was determined to 20.97%. The 1,908,234 paired reads were trimmed and assembled as previously done (Lagier et al., 2012). Extra‐genomic feature was obtained using Rast tool (Aziz et al., 2008; Brettin et al., 2015; Overbeek et al., 2014). PHAST was used for phage detection (Zhou, Liang, Lynch, Dennis, & Wishart, 2011), RNAmmer for rRNA (Lagesen et al., 2007), and Artemis was used for genome circular representation (Kumar, Tamura, & Nei, 1994). dDDH (DNA‐DNA hybridization) between the genomes was obtained using the online GGDC tool (http://ggdc.dsmz.de/ggdc.php#).

### 2.5 Phylogenetic analysis

A maximum‐likelihood 16S rRNA gene sequence‐based phylogenetic tree was constructed using the MEGA software with 500 bootstraps (Kumar et al., 1994). Blast was done (https://blast.ncbi.nlm.nih.gov/Blast.cgi?PAGE TYPE=BlastSearch), and NCBI nucleotide database (https://www.ncbi.nlm.nih.gov/nucleotide/) was used for the phylogenetically closest species with standing in nomenclatures’ sequence download. CLUSTAL W was used for sequence alignment (Thompson, Higgins, & Gibson, 1994).

## 3 RESULTS AND DISCUSSION

### 3.1 Strain Marseille‐P4678^T^ identification

**Figure 1:**
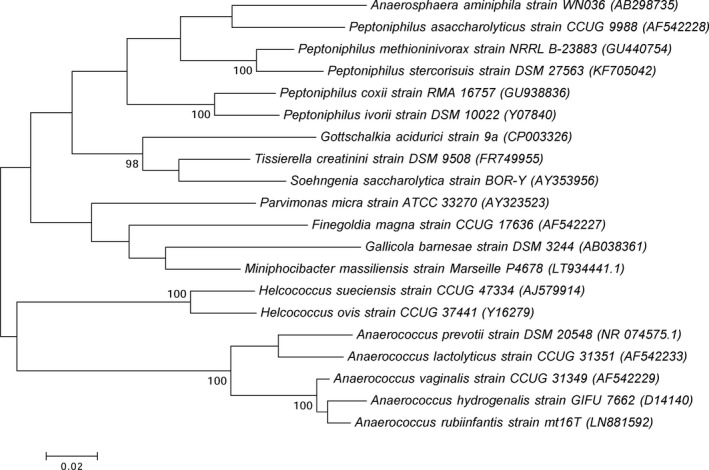
Phylogenetic tree representing the position of strain Marseille‐P4678^T^ relative to other closely related species

**Figure 2:**
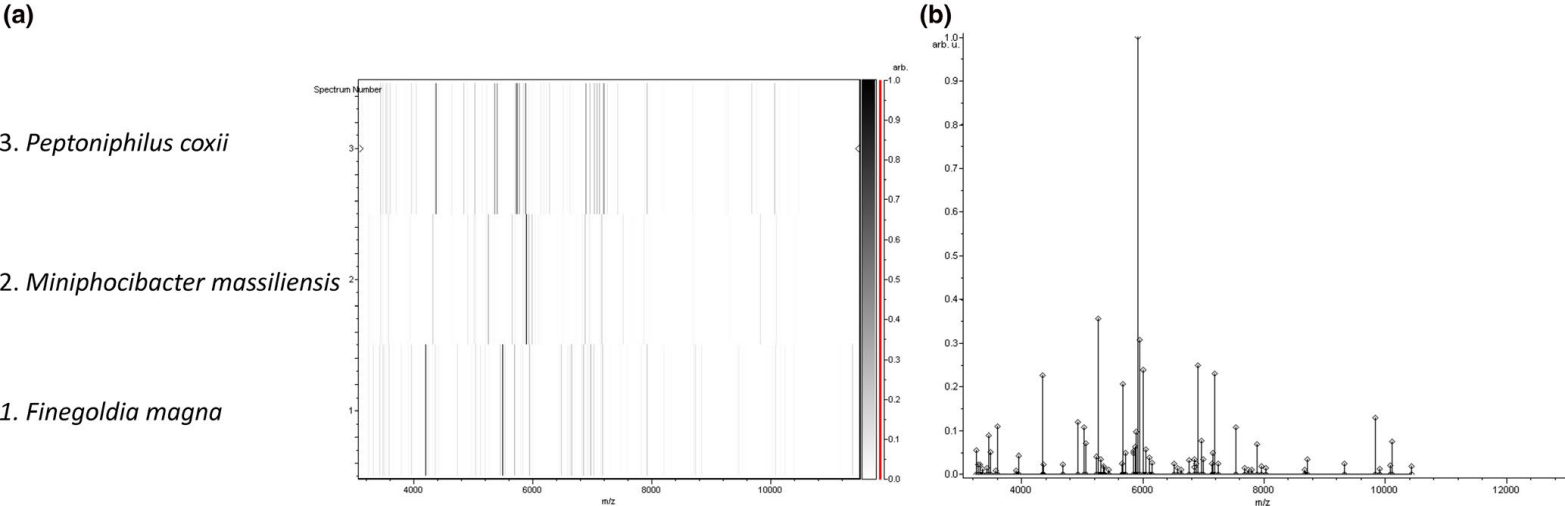
**(**a) Reference mass spectrum representing of strain Marseille‐P4678^T^ obtained after comparing 12 spectra. (b) Gel view comparing mass spectra of strain Marseille‐P4678^T^ to other species with the raw spectra on the left. The *x*‐axis represents the *m/z* value. The left *y*‐axis indicates the running spectrum number acquired from successive spectra loading. The intensity of the peaks is indicated with the different gray scale, and the *y*‐axis indicates the relation between the peak color and its intensity

The mass spectrum of strain Marseille‐P4678^T^ was missing in the current MALDI‐TOF MS Bruker database. Thus, its identification with the means of this technique was not possible. Consequently, 16S rRNA gene sequencing analysis showed that the understudied organism has more than 5% sequence divergence with *Parvimonas micra* strain 3119B^T^ (NR 036934.1), its phylogenetically closest species with standing in nomenclature (Figure 1). Thus, according to Kim et al. (2014), we propose the isolation of a new bacterial genus. To analyze at the proteomic level strain Marseille‐P4678^T^, a gel view comparing the available mass spectra of the phylogenetically closest species with standing in nomenclature was done (Figure 2b). The gel view shows that strain Marseille‐P4678^T^ has a unique peaks profile that does not match completely with any of the implemented organisms. This stands by the fact that this strain represents a new bacterial genus.

### 3.2 General features of strain Marseille‐P4678^T^

**Figure 3:**
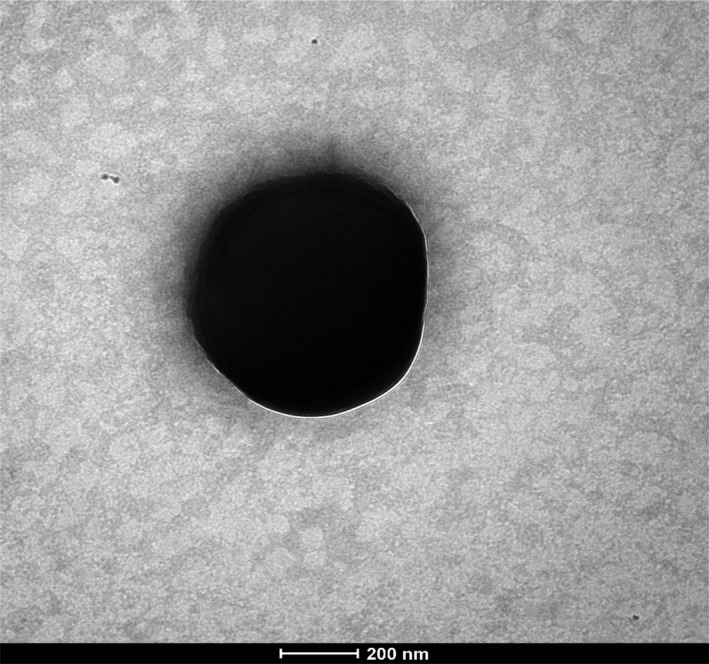
Electron micrographs of strain Marseille‐P4678^T^

Cells of this strain were Gram‐positive cocci, spore‐forming, nonmotile, catalase‐positive, and oxidase‐negative. It forms smooth gray colonies of 0.2 to 0.8 mm diameter on COS medium at 37°C after 48 hr of anaerobic incubation. Strain Marseille‐P4678^T^ cells had a diameter of 0.7 μm (Table 1, Figure 3) when observed under the electron microscope. Nevertheless, strain Marseille‐P4678^T^ grew in a temperature range between 25 and 37°C under microaerophilic and anaerobic conditions but optimally under anaerobic conditions at 37°C. This strain tolerated a pH range between 6 and 8.5 and NaCl concentration up to 100 g/L.

**Table 1:** Differential characteristics of strain Marseille‐P4678^T^, *Anaerosphaera aminiphila* (AA) (Ueki et al., 2009), *Peptoniphilus asaccharolyticus* (PA) (Ezaki et al., 2001), *Peptoniphilus coxii* (PC) (Citron, Tyrrell, & Goldstein, 2012), *Parvimonas micra* (PM) (Tindall & Euzéby, 2006), *Finegoldia magna* (FM) (Murdoch & Shah, 1999), and *Helcococcus sueciensis* (HS) (Collins, Falsen, Brownlee, & Lawson, 2004)

Properties	Strain Marseille‐P4678^T^	AA	PA	PC	PM	FM	HS
Cell diameter (μm)	0.7	0.7–0.9	Na	0.7	0.3‐0.7	0.8‐1.6	Na
Oxygen requirement	Facultative anaerobe	Anaerobic	Anaerobic	Anaerobic	Anaerobic	Anaerobic	Anaerobic
Gram stain	+	+	+	+	+	+	+
Motility	−	−	−	Na	−	−	−
Endospore formation	+	+	−	Na	−	−	−
**Production of**							
Alkaline phosphatase	−	Na	−	−	+	V	+
Catalase	+	−	Na	−	V	V	−
Urease	−	−	−	−	−	−	−
G+C content (mol%)	28.26	32.5	32.3	44.6	28.6	32.3	28.4
Habitat	Human gut	Methanogenic reactor	Clinical specimen	Clinical specimen	Human	Human	Human wound

Na: data not available; V: variable.

Strain Marseille‐P4678^T^ had its unique minimal inhibitory concentrations profile, being 0.25 > 256, 0.032, 0.032, 0.5, >256, 0.75, 0.25, 0.38, >256, 1.5, 0.25, 48, 0.064, 0.125, 0.006, 0.125, 0.25, and >32 (μg/ml) with vancomycin, amikacin, amoxicillin, benzylpenicillin, ceftriaxone, colistin, daptomycin, ertapenem, erythromycin, fluconazole, fosfomycin, imipenem, kanamycin, metronidazole, minocycline, rifampicin, teicoplanin, tobramycin, and voriconazole, respectively, proposing a possible resistance mechanism toward amikacin, colistin, kanamycin, fluconazole, voriconazole. The availability of isolating new bacterial species and drawing its antimicrobial profile urge the scientific community on pursuing efforts toward studying more exhaustively the commensal community of the human microbiota, keeping in mind that most pathogenic bacteria were previously commensals (Isenberg, 1988). Using API ZYM, positive reactions were observed for α‐galactosidase, valine arylamidase, trypsin, naphtol‐AS‐BI‐phosphohydrolase, lipase (C14), esterase lipase (C8), and cystine arylamidase. As for API 50CH, positive reactions were observed with D‐ribose, potassium gluconate, N‐acetylglucosamine, and D‐fructose. Finally, with API 20A, no positive reactions were observed. These results emphasize on the use of proteinaceous compounds as essential elements for this stain's growth. For instance, Gram‐positive anaerobic cocci bacteria are frequently isolated from human and clinical samples, and most of the latter are non‐saccharolytic and rely on peptone for growth (Ezaki et al., 2001; Ueki et al., 2009). Recently, many protein anaerobic degraders were reported such as *Clostridium thiosulfatireducens* (Hernández‐Eugenio et al., 2002), *Clostridium tunisiense* (Thabet et al., 2004), and *Proteiniphilum acetatigenes* (Chen & Dong, 2005). These bacteria were characterized by the use of proteinaceous compounds. When comparing strain Marseille‐P4678^T^ to its phylogenetically close species with standing in nomenclature, we determine that most of these species are also proteinaceous compounds users. For example, *Anaerosphaera aminiphila*, isolated from a methanogenic reactor, has been reported to be able to degrade several types of amino acids, similarly to *P. micra*,* Finegoldia*, and *Peptostreptococcu*s species, which are also member of the Gram‐positive anaerobic cocci family (Ueki et al., 2009). This highlights the potential use of strain Marseille‐P4678^T^ as protein, peptide, and amino acid degraders in the ecosystem.

General features of strain Marseille‐P4678^T^ are represented in Table 1 and are compared to its phylogenetically close species. All the included species were Gram‐positive, anaerobic, and urease‐negative (Table 1).

The major fatty acids were hexadecanoic acid (52%), 9‐octadecenoic acid (22%), and tetradecanoic acid (11%). Minor amounts of other fatty acids were also detected (Table 2). After 72 hr of culture in a hemoculture flask supplemented with blood, we measured a production of acetic (>10 mM), propanoic (3.0 ± 0.2 mM), butyric (3.5 ± 0.2 mM), isobutyric (3.0 ± 0.2 mM), isovaleric (2.2 ± 0.1 mM), and isocaproic (7.2 ± 0.3 mM) acids. Valeric, caproic, and enanthic acids were not detected.

**Table 2:** Fatty acids content of strain Marseille‐P4678^T^

Fatty acids	Name	Mean relative % ^a^
16:0	Hexadecanoic acid	51.9 ± 0.8
18:1ω9	9‐Octadecenoic acid	22.3 ± 1.4
14:0	Tetradecanoic acid	10.8 ± 1.0
18:2ω6	9,12‐Octadecadienoic acid	6.8 ± 0.4
18:0	Octadecanoic acid	2.2 ± 1.0
12:0	Dodecanoic acid	2.0 ± 0.2
15:0	Pentadecanoic acid	1.6 ± 0.4
16:1ω7	9‐Hexadecenoic acid	1.5 ± 0.1
13:0	Tridecanoic acid	TR

^a^Mean peak area percentage; TR: trace amounts <1%.

**Table 3:** Genome comparison between strain Marseille‐P4678^T^ and closely related species using GGDC and formula 2 (dDDH estimates based on identities over HSP length), upper right. The inherent uncertainty in assigning dDDH values from intergenomic distances is presented in the form of confidence intervals

	MM	PM	FM	HS	PC	PA	AA
AA	18.5 ± 2.25	18.8 ± 2.25	23.4 ± 2.35	21.6 ± 2.35	32.3 ± 2.45	19.2 ± 2.3	100%
PA	21.8 ± 2.35	24.1 ± 2.4	32.4 ± 2.45	26.8 ± 2.45	35.4 ± 2.45	100%	
PC	27 ± 2.4	28.7 ± 2.45	34.9 ± 2.45	38.7 ± 2.5	100%		
HS	27.5 ± 2.45	21.9 ± 2.35	20.4 ± 2.3	100%			
FM	20.1 ± 2.3	22.2 ± 2.35	100%				
PM	19.3 ± 2.3	100%					
MM	100%						

*Anaerosphaera aminiphila DSM 21120^T^ (AA), *Peptoniphilus asaccharolyticus* DSM 20463^T^ (PA), *Peptoniphilus coxii* DNF00729^T^ (PC), *Helcococcus sueciensis* DSM 17243^T^ (HS), *Finegoldia magna* ATCC 29328^T^ (FM), *Parvimonas micra* ATCC 33270^T^ (PM), and *Miniphocibacter massiliensis* strain Marseille‐P4678^T^ (MM).

### 3.3 Genome characteristics of strain Marseille‐P4678^T^

**Figure 4:**
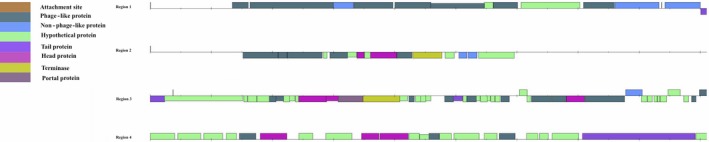
Phage like sequences distribution among the strain Marseille‐P4678^T^'s genome as predict by PHAST tool

**Figure 5:**
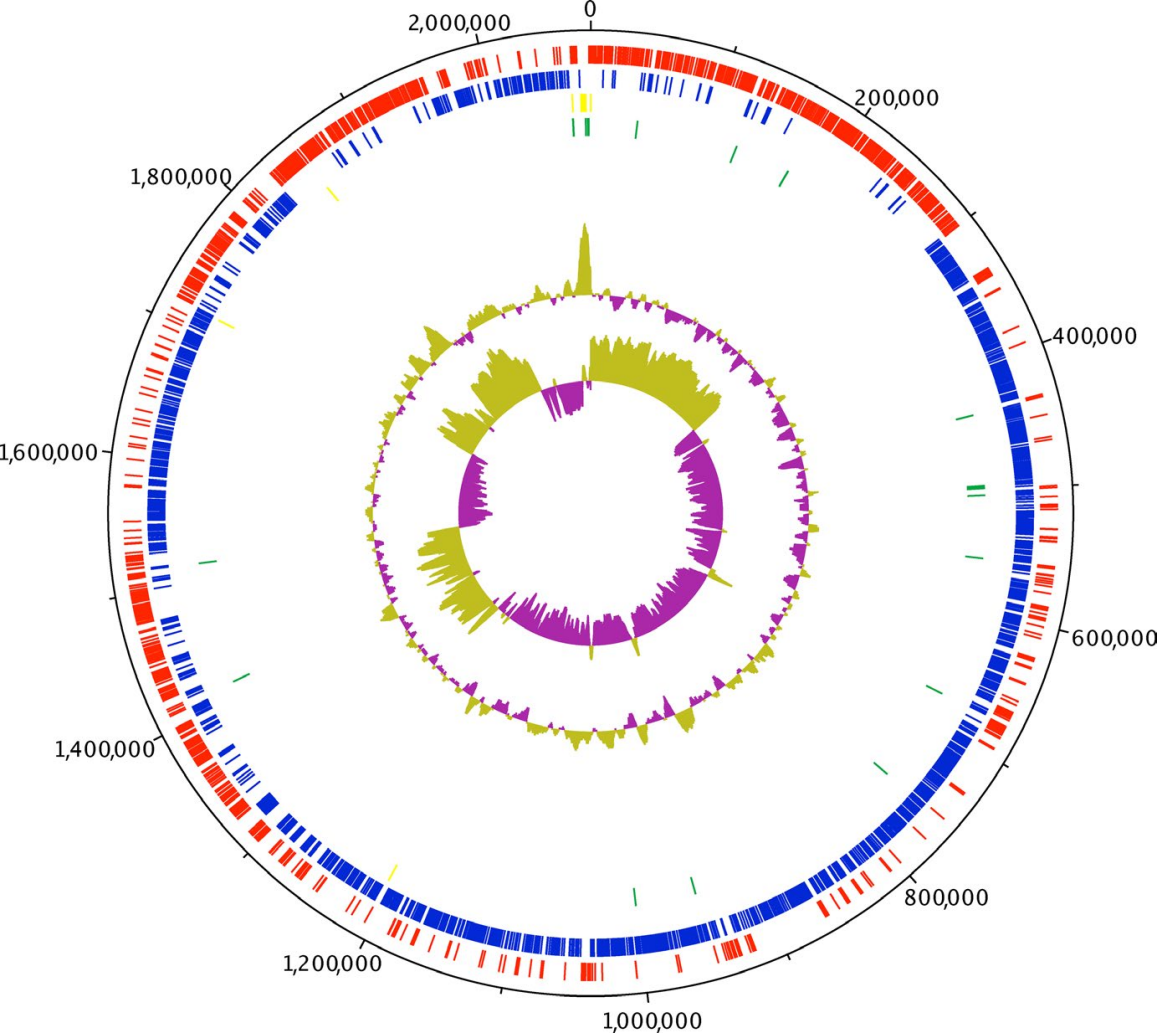
Circular representation of the strain Marseille‐P4678^T^ genome. From outer to inner: coding DNA sequences on the forward strand, coding DNA sequences on the reverse strand, rRNA, tRNA, and G+C plot and skew

The genome of strain Marseille‐P4678^T^ is 2,083,161 bp long with 28.26 mol% of G+C content. It is composed of 14 contigs. A total of 2,148 genes were detected along with 2,068 coding DNA sequences (CDSs) and 55 RNA genes. rRNA was detected as follow: 4, 3, 1 (5S, 16S, 23S) along with 44 tRNAs. No CRISPRs repeats were found. A total of 802 proteins were annotated as hypothetical proteins. Using PHAST tool, two potential prophages regions have been identified, of which one region is intact and one region is incomplete (Figure 4). The intact region extended in the region 1012948–1042003 with 29.88 mol% of G+C content and shared the highest number of proteins with *Bacillus* virus 1 (NC 009737) with 25% similar proteins. Phage tracking is important since they might be contributing to virulence mechanisms acquiring due to gene transfer events (Penadés, Chen, Quiles‐Puchalt, Carpena, & Novick, 2015). Based on RAST annotation, 25 factors were correlated to virulence, diseases, and defense from which 12 were correlated to antibiotics resistance and toxic compounds (copper homeostasis, Million et al., 2016, cobalt‐zinc‐cadmium resistance, Vétizou et al., 2015, aminoglycoside adenylyltransferases, Clemente et al., 2012, fluoroquinolones resistance, Million et al., 2016, cadmium resistance, Clemente et al., 2012, and multidrug resistance pumps, Million et al., 2016). As well, 12 invasion and intracellular resistance features were detected (*Mycobacterium* virulence operon involved in protein synthesis (SSU ribosomal proteins) (Rigottier‐Gois, 2013), *Mycobacterium* virulence operon involved in an unknown function with a Jag Protein and YidC and YidD (Million et al., 2016), *Mycobacterium* virulence operon involved in DNA transcription (Million et al., 2016), and *Mycobacterium* virulence operon involved in protein synthesis (LSU ribosomal proteins) (Tidjani Alou et al., 2017). Spore protection system features were detected, and no motility features were detected, confirming our previous results. Circular genome representation of strain Marseille‐P4678^T^ is shown in Figure 5.

### 3.4 Comparative analysis between the genomes of strain Marseille‐P4678^T^ and closely related species

GGDC online calculator was used with formula 2, in order to calculate the DNA‐DNA hybridization distance (dDDH) between the genome of strain Marseille‐P4678^T^ and other available genomes of the phylogenetically closest species (Table 3). dDDH values are calculated whenever whole genome sequence is available in order to confirm other proteomic, phenotypic, and genomic data that propose the classification of a new species (Mccarthy & Bolton, 1963; Schildkraut, Marmur, & Doty, 1961). Being set as a norm, 70% threshold is adapted to delimitate a species (Mccarthy & Bolton, 1963; Schildkraut, Marmur, & Doty, 1961). Strain Marseille‐P4678^T^ had dDDH values of 18.5, 21.8, 27, 27.5, 20.1, and 19.3 with *Anaerosphaera aminiphila* WN036^T^ (*A. aminiphila*), *Peptoniphilus asaccharolyticus* ATCC 14963^T^ (*P. asaccharolyticus)*,* Peptoniphilus coxii* RMA 16757^T^ (*P. coxii)*,* Helcococcus sueciensis* CIP 108183^T^ (*H*. *sueciensis*), *Finegoldia magna* DSM 20470^T^ (*F. magna)*, and *Parvimonas micra* ATCC 33270^T^ (*P. micra*), respectively, thus supporting the previous data that suggest the classification of this as a novel bacterial species (Table 3).

The draft genome sequence of strain Marseille‐P4678^T^ size is lower than *Peptostreptococcus assachrolyticus* but higher than *H. sueciensis*,* P. micra, F. magna*,* and P. coxii* (2.1, 2.2, 1.6, 1.6, 1.8, and 1.8, respectively). The G+C content (mol%) of strain Marseille‐P4678^T^ is higher than those of *H. sueciensis* but lower than *P. micra, F. magna, P. asaccharolyticus, A. aminiphila*, and *P. coxii* (28.26, 28.4, 28.6, 32.3, 32.3, 32.5, and 44.6, respectively). CDSs in strain Marseille‐P4678^T^ were higher than *H. sueciensis, P. micra, P. coxii, F. magna, A. aminiphila*, and *P. asaccharolyticus* (2134, 1427, 1476, 1742, 1839, 1,916, and 2054).

## 4 CONCLUSION

Culturomics has proved that culture is an essential method that should be adapted in complementarity with metagenomics when describing the human microbiota and especially the gut. With the means of this approach, we succeeded in isolating a new bacterial genus (*M. massiliensis* strain Marseille‐P4678^T^ gen. nov. sp. nov.), thus adding to the current human gut repertoire new species. Its taxono‐genomics description rendered its main features available for the scientific community in case of its isolation in a commensal or pathogenic scene. Nevertheless, sequencing its genome made more nucleotides sequences defined and thus minimized the holes in the current database or what is so‐called Dark Matter.

### 4.1 Description of *Miniphocibacter* gen. nov

*Miniphocibacter* (Mini.phoci.bacter, L. adj. masc., *miniphocibacter* composed by *mini*, referring at the small size of pygmy people from whom this strain was isolated, and *phoci* referring at Phocae, the Latin name of the city from where the funders of culturomics came from). Cells are Gram‐positive cocci, spore‐forming, non‐motile, catalase‐positive, and oxidase‐negative. It forms smooth gray colonies of 0.2 to 0.8 mm diameter on COS medium at 37°C after 48 hr anaerobic incubation and grows optimally under anaerobic conditions at 37°C. Major fatty acids were hexadecanoic acid (52%), 9‐octadecenoic acid (22%), and tetradecanoic acid (11%). Minor amounts of other fatty acids were also detected. The genome of strain Marseille‐P4678^T^ is 2,083,161 bp long with 28.26 mol% of G+C content. The type species is *M. massiliensis*.

### 4.2 Description of *M. massiliensis* sp. nov

*Miniphocibacter massiliensis* (mas.il.i.en'sis, L. gen. masc. n., *massiliensis*, pertaining to Massilia, the antic name of the city of Marseille, where this bacterium was discovered).

Cells are Gram‐positive cocci with 0.7 μm in diameter, spore‐forming, non‐motile, catalase‐positive, and oxidase‐negative. It forms smooth gray colonies of 0.2 to 0.8 mm diameter on COS medium at 37°C after 48 hr anaerobic incubation. Nevertheless, strain Marseille‐P4678^T^ grows in a temperature range between 25 and 37°C under microaerophilic and anaerobic conditions but optimally under anaerobic conditions at 37°C. It tolerates a pH range between 6 and 8.5 and NaCl concentration more than 100 g/L. Using APIZYM, positive reactions are observed for α‐galactosidase, valine arylamidase, trypsin, naphtol‐AS‐BI‐phosphohydrolase, lipase (C14), esterase lipase (C8), and cystine arylamidase. As for API50CH, positive reactions are observed with D‐ribose, potassium gluconate, N‐acetylglucosamine, and D‐fructose. Finally, with API20A, no positive reactions are observed. Major fatty acids are hexadecanoic acid (52%), 9‐octadecenoic acid (22%), and tetradecanoic acid (11%). Minor amounts of other fatty acids are also detected. The genome of strain Marseille‐P4678^T^ is 2,083,161 bp long with 28.26 mol% of G+C content.

The type strain is Marseille‐P4678^T^ and was isolated from the stool sample of a healthy 39‐year‐old pygmy male from Congo.

## CONFLICT OF INTEREST

None to be declared.

## AUTHOR CONTRIBUTIONS

MB isolated, described, and wrote the manuscript. MF helped in the taxono‐genomics description. TN helped in the genomic sequencing. MR helped in genome sequencing. ZD helped in writing and critical analysis of the manuscript. PF helped in writing and critical analysis of the manuscript. DR designed the project, and helped in writing, reviewing, and critical analysis. FC designed the study, analyzed the data, and wrote the manuscript.

## ETHICS STATEMENT

An approval under the number 09‐022 was obtained from the Ethic Committee of the Institut Fédératif de Recherche 48 along with a signed consent from the sample's donor. The donor was a healthy 39‐year‐old pygmy male, and collection was done according to Nagoya protocol from Republic of Congo.

## Data Availability

Genome sequences of strain Marseille‐P4678^T^ were deposited in EMBL‐EBI and can be accessed with the following accession numbers: LT934441 and OCTQ00000000. The type strain is Marseille‐P4678^T^ and was deposited in two international strain collection institutes with the following accession numbers: CSUR P4678^T^ = CCUG 71375^T^. Strain Marseille‐P4678^T^ typical mass spectrum can be accessed on http://www.mediterranee-infection.com/article.php?larub=280&titre=urms-database.
