# EXPRESSION OF CONCERN: *Miniphocibacter massiliensis* gen. nov., sp. nov., A New Species Isolated from the Human Gut and Its Taxono‐Genomics Description

**DOI:** 10.1002/mbo3.70248

**Published:** 2026-02-19

## Abstract

**EXPRESSION OF CONCERN:** M. Bilen, M.D.M. Fonkou, T.T. Nguyen, M. Richez, Z. Daoud, P‐E. Fournier, D. Raoult, and F. Cadoret, *“Miniphocibacter massiliensis* gen. nov., sp. nov., A New Species Isolated from the Human Gut and Its Taxono‐Genomics Description,” *Microbiology Open* 8, no. 5 (2019): e00735, https://doi.org/10.1002/mbo3.735.

This Expression of Concern is for the above article, published online on 2 October 2018 in Wiley Online Library (wileyonlinelibrary.com), and has been issued by John Wiley & Sons Ltd. The Expression of Concern has been agreed due to questions raised about the study's adherence to French legal and ethical requirements for research involving human subjects, including questions regarding proper informed consent for research involving vulnerable populations. A concern has also been raised by a third party about a potential conflict of interest for co‐author for Didier Raoult. The investigation into these concerns is ongoing. Therefore, the journal has decided to issue an Expression of Concern to inform and alert readers.

